# Implementation and evaluation of a complex outpatient oral antimicrobial therapy program (COpAT) in Canada

**DOI:** 10.1017/ash.2025.19

**Published:** 2025-02-12

**Authors:** Maggie Wong, Davie Wong

**Affiliations:** 1 Fraser Health Authority, University of British Columbia, Canada; 2 Department of Pharmacy, Royal Columbian Hospital, New Westminster, BC, Canada; 3 Division of Infectious Diseases, Department of Medicine, Royal Columbian Hospital, New Westminster, BC, Canada

## Abstract

**Objective:**

We describe the implementation, outcomes, and challenges of a complex outpatient oral antimicrobial therapy program (COpAT) in Canada to provide a framework for those interested in establishing such a program.

**Setting:**

Outpatient ambulatory clinic led by infectious diseases physicians, serving patients from a tertiary hospital and a small community hospital.

**Design:**

Retrospective observational study that evaluated the efficacy, safety, and cost savings of patients enrolled in the program from August 2023 to June 2024.

**Results:**

One hundred three patients were included, of which 84.4% achieved successful clinical outcomes. Mean age of the patients was 62 years and 30% had diabetes. The top three sources of infections were bone and joint, intra-abdominal, and skin-and-soft tissue. Mean duration of COpAT was 37 days. Seventy-five percent of patients required only a single agent, and amoxicillin/clavulanic acid was most commonly used. Twenty-two patients developed an adverse reaction, of which three required a change in therapy and one resolved with antibiotic dose reduction. No C. difficile infections or mortality were reported 30-days post COpAT discharge. Twelve patients were re-admitted to the hospital; 50% of the cases were unrelated to infections. Compared to outpatient intravenous therapy, the total cost savings from COpAT were estimated to be $255,000 Canadian dollars (CAD), which translated to an average cost savings of $2500 CAD per patient per year.

**Conclusion:**

We demonstrated favorable clinical and safety outcomes with our COpAT program and substantial cost savings using existing infrastructure. COpAT allows efficient use of healthcare resources including decongestion of hospitals.

## Background

Outpatient parenteral program, defined as a home intravenous program or therapy given at an infusion center, has been used for the last few decades as an effective and safe method of administering antimicrobials to patients.^1^ While intravenous (IV) therapy may be thought to be better than their oral counterparts, there are numerous recent randomized controlled studies demonstrating the non-inferiority of oral antibiotic regimens for infections such as osteomyelitis,^2^ endocarditis,^3^ and bacteremia.^4–6^ In addition, complications such as venous thrombosis associated with line insertion and excessive costs associated with parenteral therapy can be avoided altogether.^7^

The implementation of complex outpatient oral antimicrobial therapy clinic (COpAT) has been described in the United Kingdom (UK) and the United States,^8,9^ but it is a novel concept in Canada. The definition of COpAT varies in the literature; it is generally considered complex when a patient requires prolonged treatment of antimicrobial(s) that can potentially cause significant side effects.^9^ We sought to describe our experience in setting up a COpAT program at a large tertiary hospital and a smaller community hospital in British Columbia, Canada. We also evaluated the outcomes of our COpAT program, including efficacy and safety for patients, and cost savings. This could serve as a guide for design and human resource allocation for other centers that would like to implement COpAT.

## Methods

This is a retrospective observational study that described the implementation of COpAT at 2 hospitals with existing home intravenous (home IV) and outpatient parenteral antimicrobial therapy (OPAT) programs, and evaluated the associated outcomes.

### Setting

The first site is a 450-bed tertiary hospital which specializes in services such as trauma, cardiac surgery, and neurosurgery. The second site is a 170-bed community hospital that provides inpatient and outpatient care in general medicine, surgery and other specialty services such as urology, plastics, and orthopedics. These 2 sites are grouped together for our COpAT implementation since they belong to the geographical areas served by the same regional infectious diseases physician (ID) group.

### Description of the existing programs and infrastructure

There are five ID physicians who serve both sites through rotation every 2 weeks. They oversee the home IV program, OPAT and outpatient ID clinic. The home IV program is supported by a full-time home IV nurse and a unit clerk. Patients in the home IV program learn how to self-administer IV antimicrobials at home, whereas those who are unable to do so or require less than 1 week of IV treatment would go to OPAT daily to receive treatment given by nurses. The OPAT clinics are infusion centers located in the ambulatory care department at both hospitals. At OPAT, all patients are usually reviewed by an ID physician every 5–14 days depending on their complexity to determine the need for ongoing IV treatment, whether to switch to oral antimicrobial(s) or discontinue antibiotic therapy altogether. Patients can also be referred to the outpatient ID clinic for evaluation of an infectious diagnosis and determine the need for antimicrobials (IV or oral). The ID physician will then refer patients to the home IV program, the OPAT clinic or COpAT accordingly.

The COpAT program shares the same human resources as the home IV program and is located in the same building as OPAT. There is no designated pharmacist for home IV, OPAT or the outpatient ID clinic; they would only assist with drug level monitoring (eg, vancomycin, aminoglycosides) on demand.

### COpAT enrollment criteria

A safe and effective oral regimen is available. The patients need to be clinically stable as per the ID physician, and require at least 2 weeks of oral antimicrobials. They must be able to absorb and tolerate oral medication, have no psychosocial reason to prefer IV treatment, and can afford therapy. To minimize the failure of oral therapy, source control is either not required or considered to be adequate per the ID physician.

### Process of referral to COpAT and follow-up

The referral source of patients can be from an inpatient unit, OPAT, or the outpatient ID clinic. Once a patient is referred to COpAT by the ID physician, a home IV nurse facilitates patient enrollment. She provides the patient with a lab requisition for weekly bloodwork and books a follow-up appointment with the physician. Frequency of follow-up is determined by the ID physician, usually every 2–4 weeks. The follow-up appointments can be in-person or via telehealth.

### Timeline

**Phase 1**: August 2023 to June 2024 (the first COpAT patient was enrolled in August 2023). During this phase, all COpAT patients were followed by the ID physicians.

**Phase 2**: In July 2024, an existing full-time antimicrobial stewardship (AMS) pharmacist was recruited to assist with the monitoring of all COpAT patients who resided in the area of the tertiary site. The home IV nurse would notify her once the patient was enrolled in COpAT. Since this was a pilot project, no additional funding was requested to support the COpAT program.

### Data collection and outcomes evaluation

Data collection was done by retrospective chart review from August 1, 2023 to June 30, 2024 using the Meditech electronic health record. Patient demographics (age, sex, comorbidities), indications and duration of oral antimicrobial(s) while in COpAT plus duration of prior IV therapy if applicable, and bacterial culture(s) identified were collected for all included patients.

Primary outcomes included clinical improvement, and allergic or adverse reactions to oral antimicrobials including *C. difficile* infection. Clinical success was defined as no further antimicrobials needed at the end of treatment. Partial response was defined as switching to another oral agent (eg, due to side effects) or extending duration of therapy for the same infection. Failure was defined as transitioning to IV therapy for any reason. Secondary outcomes included 30-day readmission and 30-day mortality rates post discharge from the COpAT program, loss to follow-up, and potential cost savings by COpAT enrollment compared to the home IV program. Primary and secondary outcomes were reported using descriptive statistics. Since this was a retrospective descriptive study, it was not subject to local health authority ethics board review.

### Cost analysis

The cost savings was calculated by taking the difference between the cost of an appropriate hypothetical home IV regimen and the actual oral therapy that was prescribed. The prices of the relevant oral and IV medications were obtained from public and hospital databases. The cost of home IV supplies was obtained from an outpatient pharmacy contracted by our hospitals to deliver and provide the necessary equipment for setting up IV treatment at home. The cost of a central venous catheter was provided by our home IV program. Although the use of oral antimicrobials is associated with earlier hospital discharge and completely obviates any central line related complications, these parameters were not assessed in our study, but would likely contribute to additional unmeasured cost savings. In our institutions, home IV therapy is 100% publicly funded while oral treatment is not. Therefore, the money and resources saved from prescribing oral therapy benefit only the hospital.

## Results

One hundred and twelve patients were screened, of which 103 were included. Reasons for exclusion were listed in Table 1. Seventy-eight percent of enrolled patients came from the community hospital. The inpatient service at the sites generated 48% of the referrals. The mean age was 62 years, with type 2 diabetes being the most common comorbidity, followed by cardiovascular diseases and cancers. The top three indications were bone and joint (48%), intra-abdominal (17%), and skin and soft tissue infections (11%). The median duration of IV therapy (prior to COpAT) and oral antimicrobial(s) once in the program were 7 days and 31 days, respectively. Twelve patients received only oral antimicrobial(s) for the entire treatment duration. Seventy five percent of patients required a single-agent; amoxicillin/clavulanate acid (56%) was the most commonly used agent, followed by doxycycline (23%). See Table 1 for most commonly identified bacterial cultures and intravenous agents used prior to oral step down.

**Table 1.** Demographics and treatment regimens of included patients

Total number of screened patients	112
Tertiary hospital	23
Community hospital	80
Excluded patients	9
Reasons for exclusion (number of patients)	Had less than 14 days of oral antibiotic(s) (2)
	On home IV program (2)
	Already finished antibiotic(s) by the time patient was seen by ID physician and no further treatment needed (2)
	Maintained on IV therapy and never left the hospital (1)
	ID physician didn’t think the patient has an infection at follow-up appointment (1)
	Lost to follow-up (1)
Total number of included patients	103
Source of referral (% of patients)	Hospital: 49 (48)
	Outpatient parenteral antimicrobial therapy program: 34 (33)
	Outpatient ID clinic: 20 (19)
Age (years)	Mean: 62 years
Sex (% of patients)	Male: 62 (60)
	Female: 41 (40)
Comorbidities N = 103 (% of patients)	
Diabetes	31 (30)
Cardiovascular conditions	16 (16)
Note: Patient can have more than 1 cardiac condition	Stroke: 3
	Myocardial infarction: 10
	Congestive heart failure: 4
Peripheral vascular disease	7 (7)
COPD	9 (9)
Liver disease	1 (1)
Moderate to severe chronic kidney disease	15 (15)
Cancer	16 (16)
Note: Patient can have more than 1 type of cancer	Solid: 15
	Hematologic: 2
Immunosuppressed	8 (8)
Types of infection (% of patients)	Bone and joint infections: 49 (48)
	Intra-abdominal: 18 (17)
	Skin and soft tissues: 11 (11)
	Dental/ear/nose/throat: 6 (6)
	Respiratory infections: 5 (5)
	Endovascular: 5 (5)
	Urinary tract: 5 (5)
	Others (e.g more than 1 type of infections): 4 (4)
Duration of IV antimicrobial (prior to COpAT enrollment)	11 days (mean)
	7 days (median)
Duration of oral antimicrobial during COpAT	37 days (mean)
	31 days (median)
IV antimicrobial regimen prior to oral stepdown n = 91 (% of patients)	Ceftriaxone: 58 (64)
Note: 12 patients didn’t require any IV antimicrobial, and each patient can be on more than 1 agent	Cefazolin: 17 (19)
	Carbapenem: 10 (11)
	Daptomycin: 10 (11)
	Piperacillin/tazobactam: 7 (8)
	Amoxicillin/clavulanic acid: 7 (8)
	Vancomycin: 5 (5)
Oral antimicrobial regimen	Single agent: 77 (75)
N = 103 (% of patients)	Greater than 1 agent: 26 (25)
	**Antimicrobial(s) used**:
	Amoxicillin/clavulanic acid: 58 (56)
	Doxycycline: 24 (23)
	Quinolones: 15 (15)
	Amoxicillin: 11 (11)
	Cephalosporins: 10 (10)
	Metronidazole: 4 (4)
	Linezolid: 3 (3)
	Trimethoprim/Sulfamethoxazole: 3 (3)
	Rifampin: 3 (3)
	Clindamycin: 2 (2)
Bacterial culture most commonly identified	**Gram positive pathogens**
N = 103 (% of patients)	Methicillin-susceptible *S. aureus*: 25 (24)
	Methicillin-resistant *S. aureus*: 4 (4)
	Streptococcus species: 25 (24)
	**Gram negative pathogens**
	*E. coli*: 8 (8)
	*K. pneumoniae*: 6 (6)

Abbreviations: IV, intravenous; ID, infectious diseases; COPD, chronic obstructive pulmonary disease; COpAT, complex outpatient oral antimicrobial therapy.

Regarding primary outcomes, 87 patients (84.4%) completed therapy successfully. Ten patients (10%) experienced clinical failure for various reasons, such as needing additional source control or having a different infection. Twenty-two patients (21%) developed an adverse reaction to oral antimicrobial(s) and 1 patient developed an allergic reaction; as a result, 5 patients (4.8%) required a change in therapy (See Table 2 for details). The rest of these patients experienced minor side effects, but were able to continue treatment. No cases of *C. difficile* infection were reported. There was no loss to follow-up. Twelve patients (12%) were readmitted to the hospital within 30 days of stopping oral therapy, but half of them were unrelated to infections; no deaths occurred during the same period. See Table 2 for summary of outcomes.

**Table 2.** Summary of primary and secondary outcomes

Primary outcomes N = 103 (% of patients)	
Clinical success	87 (84.4)
Partial response	6 (5.8)
Treatment failure	10 (9.7)
Adverse reactions to antimicrobials	22 (21)
Note that a patient can have more than 1 type of adverse reactions	Diarrhea: 10
	Nausea: 6
	Others: 8
Allergic reactions to antimicrobials	1 (1)
Change in therapy due to adverse or allergic reaction (s)	5 (5)
Reasons for change in therapy (antimicrobial involved, outcome after treatment change)	Nausea and weakness (rifampin, resolved after switching to cephalexin)
	Thrombocytopenia (linezolid, resolved after switching to moxifloxacin)
	Nausea and gastrointestinal side effect (doxycycline, resolved after switching to levofloxacin)
	Back rash (cefuroxime, resolved after stopping)
	Diarrhea (cephalexin, resolved with dose reduction)
**Secondary outcomes N = 103 (% of patients)**	
30-day readmission rate	12 (11.6)
	Related to infection: 6
	Unrelated to infection: 6
30-day mortality rate	0
Loss to follow-up	0

A cost comparison analysis was performed to evaluate the cost savings of the COpAT program. The total expenditure for oral therapy among the 103 patients was just over $15,000 CAD. The total cost of an equivalent home IV regimen for all these patients was estimated at over $270,000 CAD, a difference of $255,000 CAD. This amounts to an average cost savings of $2500 CAD per patient per year. The cost savings represented a 94% reduction from the total cost of intravenous antibiotics combined with supplies.

## Discussion

To our knowledge, this is the first study describing an implementation of COpAT program in Canada. Traditionally, osteomyelitis, diabetic foot and prosthetic joint infections are treated with IV therapy, so resources were placed in OPAT or home IV to facilitate hospital discharge. These patients made up almost 60% of the patient population seen by our ID physicians. Similar to the OVIVA study^2^ and a subsequent implementation study in the UK,^8^ our results showed that patients who met the inclusion criteria can be treated successfully with oral antimicrobials; our clinical success and 30-day hospital readmission rate were similar to those achieved by outpatient parenteral therapy.

From an AMS standpoint, transition to oral therapy may help narrow the spectrum of antimicrobials. Twenty two percent of our patients were on carbapenems and daptomycin prior to oral step down; the World Health Organization considers these agents as last-line therapy, and they should be reserved for multi-drug resistant organisms. On the other hand, our most commonly used antibiotics, amoxicillin/clavulanic acid and doxycycline, are on the “access” list which have lower potential for resistance and side effects compared to other agents.^10^ Compared to OPAT, where once daily antibiotics, such as daptomycin or ertapenem, are chosen for convenience, COpAT has the added advantage of sparing these agents without compromising efficacy in selected patients.

In our practice, there is no minimum duration of IV lead in before transitioning to oral therapy. The decision to switch to oral treatment is based on clinical judgment by the ID physician after carefully considering the patient’s severity of illness and clinical status, type of infection, the degree of source control if applicable, and any relevant literature.

For this pilot project, no additional funding or human resources were required; we engaged the existing resources (eg, home IV nurse and infrastructure) to help with patient enrollment into the COpAT program. However, with increased demand and program expansion, appointment time slots for ID physician to review patients could fill up quickly on certain days of the week. To overcome this barrier, we are experimenting with having an AMS pharmacist follow some of the enrolled patients via phone call. This model is based on studies demonstrating telemedicine as successful delivery of OPAT.^11^

Twenty-two percent of our patients developed an adverse or allergic reaction to the oral antimicrobials. Majority of these reactions occurred in patients on dual therapy, or higher-risk agents such as linezolid. Therefore, the AMS pharmacist can place a higher priority on these patients (25% of the enrolled patients). This may help offload the need for ID physicians to reassess all COpAT patients on a weekly to bi-weekly basis, which in turn can free up clinic spots for additional patients. ID physicians will continue to reassess all patients at the end of treatment.

Oral therapy is almost always less expensive than IV treatment. In this study, we demonstrated a substantial cost savings over a one-year period, which is consistent with results from other studies.^12^ The money saved could be used toward expanding COpAT and/or securing more resources to optimize its function and flow.

Aside from the aforementioned cost benefits, oral treatment affords higher convenience for patients, avoids the need for vascular access and its associated complications, offers higher cost-effectiveness, facilitates earlier discharge from hospital, minimizes staff workload required to prepare and administer IV medications, and leaves a smaller carbon footprint compared to its IV counterpart.^13,14^ While patient satisfaction is not formally measured in our study, patients invariably choose oral options over IV therapy, whether it is given via home IV or OPAT, if given a choice by the ID physician. A small patient survey done in the UK found similar results.^15^

A strength of this study is that patients with a wide variety of infections from 2 different sites were included, which improves generalizability to other centers. Some limitations of this study include its retrospective nature and the short duration of follow-up (30 days) after stopping oral therapy; it is possible that treatment failure occurs after that. The cost comparison analysis only measured the costs of medications and supplies. The cost of home health services, complications related to vascular access, and days in hospital were not factored into the calculations. Therefore, the true cost savings are likely to be higher.

Despite these limitations, our results suggest that from efficacy, safety, and cost standpoints, COpAT offers a useful and sustainable alternative to OPAT and home IV program for patients who meet inclusion criteria. It also helps to free up inpatient beds for other activities, which is of utmost importance to improve utilization of limited hospital resources.

We were able to successfully implement a COpAT program at 2 Canadian hospitals using the existing human resources and infrastructure of ID led outpatient IV antibiotic programs. We demonstrated that clinical and safety outcomes of oral antibiotic regimens were similar to the outpatient parenteral program literature, but with substantial cost savings. For patients who meet enrollment requirements, COpAT is an attractive option, and our program will continue to expand to other sites in the near future.
